# Development of dermatomyositis after anti-transcriptional intermediary factor 1-γ antibody seroconversion during treatment for small cell lung cancer

**DOI:** 10.1186/s12890-022-01974-4

**Published:** 2022-05-12

**Authors:** Yuki Sato, Yoshinori Tanino, Takefumi Nikaido, Ryuichi Togawa, Takaya Kawamata, Natsumi Watanabe, Riko Sato, Ryuki Yamada, Takumi Onuma, Hikaru Tomita, Mikako Saito, Mami Rikimaru, Julia Morimoto, Yasuhito Suzuki, Hiroyuki Minemura, Junpei Saito, Kenya Kanazawa, Syoki Yamada, Yuko Hashimoto, Yoko Shibata

**Affiliations:** 1 Department of Pulmonary Medicine, School of Medicine, Fukushima Medical University, 1 Hikarigaoka, Fukushima, 960-1295 Japan; 2 Department of Diagnostic Pathology, School of Medicine, Fukushima Medical University, 1 Hikarigaoka, Fukushima, 960-1295 Japan

**Keywords:** Cancer-associated myositis, Anti-TIF1-γ antibody, Seroconversion, Case report

## Abstract

**Background:**

Inflammatory myositis, such as dermatomyositis, is sometimes complicated by cancer and is recognized as cancer-associated myositis. Although some autoimmune antibodies are considered to be involved in the development of myositis in cancer patients, the precise mechanism has not been clarified. The findings of the present case shed light on the mechanism by which anti-transcriptional intermediary factor 1 (TIF1)-γ Ab was produced and the pathogenesis of cancer-associated myositis.

**Case presentation:**

We describe a case of dermatomyositis that developed in a 67-year-old man who had been diagnosed with small cell lung cancer of clinical T4N3M0 stage IIIB/limited disease during treatment. He received systemic chemotherapy and radiation therapy, and dermatomyositis developed along with a significant decrease in tumor size. TIF1-γ Ab, which is one of the myositis-specific antibodies, was found to be seroconverted. In addition, immunohistochemical analysis showed that cancer cells were positive for the TIF1-γ antigen.

**Conclusion:**

The findings of the present case suggest that transcriptional intermediary factor 1-γ, which is released from tumor cells, induces the production of TIF1-γ Ab, leading to the development of dermatomyositis.

**Supplementary Information:**

The online version contains supplementary material available at 10.1186/s12890-022-01974-4.

## Background

Idiopathic inflammatory myopathies such as polymyositis and dermatomyositis have been reported to be associated with malignant tumors [1, 2]. In patients with cancer-associated myositis (CAM), anti-transcriptional intermediary factor 1 (TIF1)-γ Ab is frequently found in the serum, suggesting a relationship between the antibody and CAM [3]. However, the mechanism by which TIF1-γ Ab is involved in the pathogenesis of CAM has yet to be clarified. In this report, we describe the development of dermatomyositis accompanied by TIF1-γ Ab seroconversion after cancer therapy in a patient with small cell lung cancer. Immunohistochemical analysis demonstrated that tumor cells expressed the TIF1-γ antigen. These findings suggest that the release of antigens from tumor cells is involved in TIF1-γ Ab seroconversion, which causes the development of dermatomyositis.

## Case presentation

**Fig. 1:**
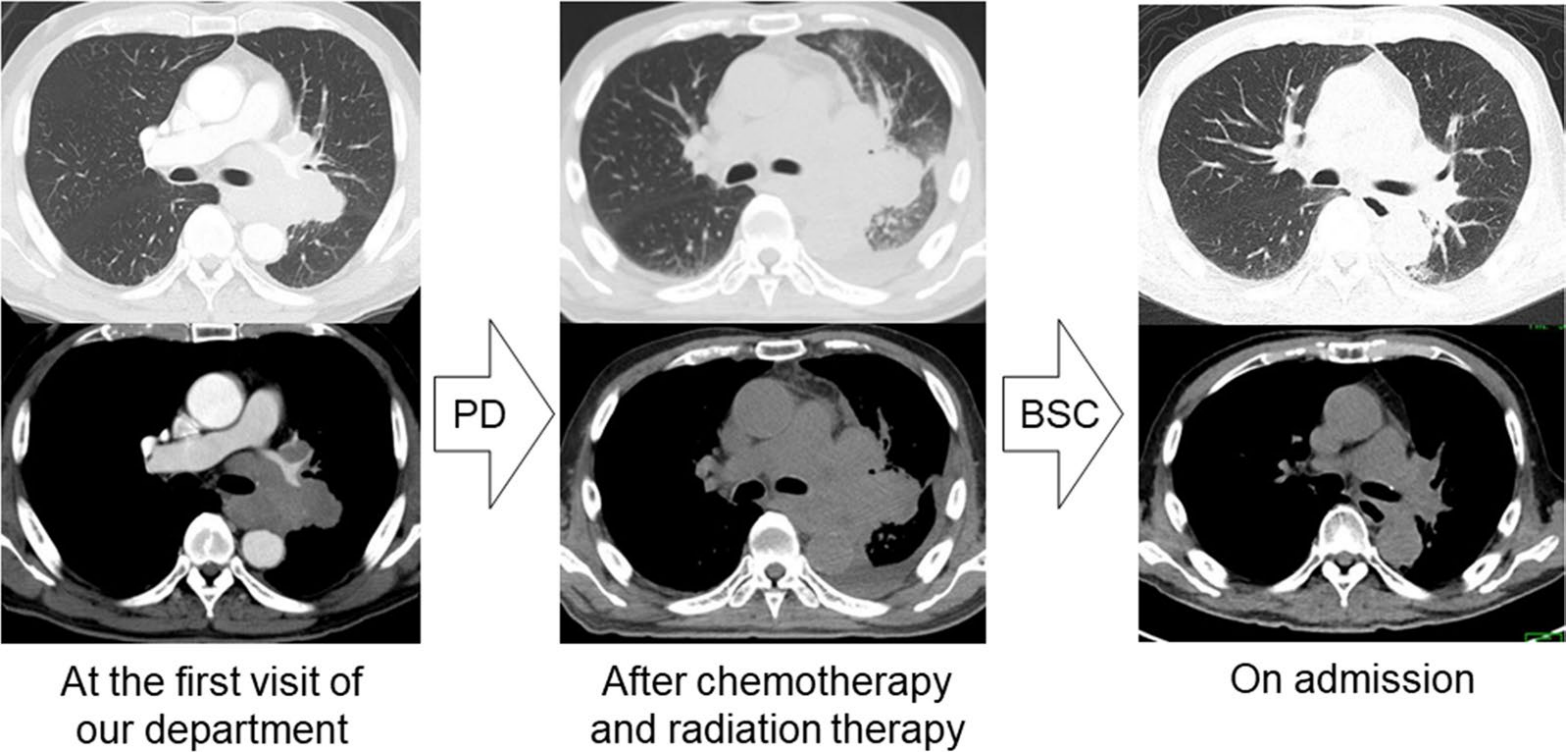
A tumor shadow was observed in the left hilum during the first visit to our department. Chemotherapy and radiotherapy were performed but terminated due to a decrease in performance status. The patient received no aggressive therapy, but the size of the tumor gradually decreased over one month. PD, progressive disease; BSC, best supportive care

**Fig. 2:**
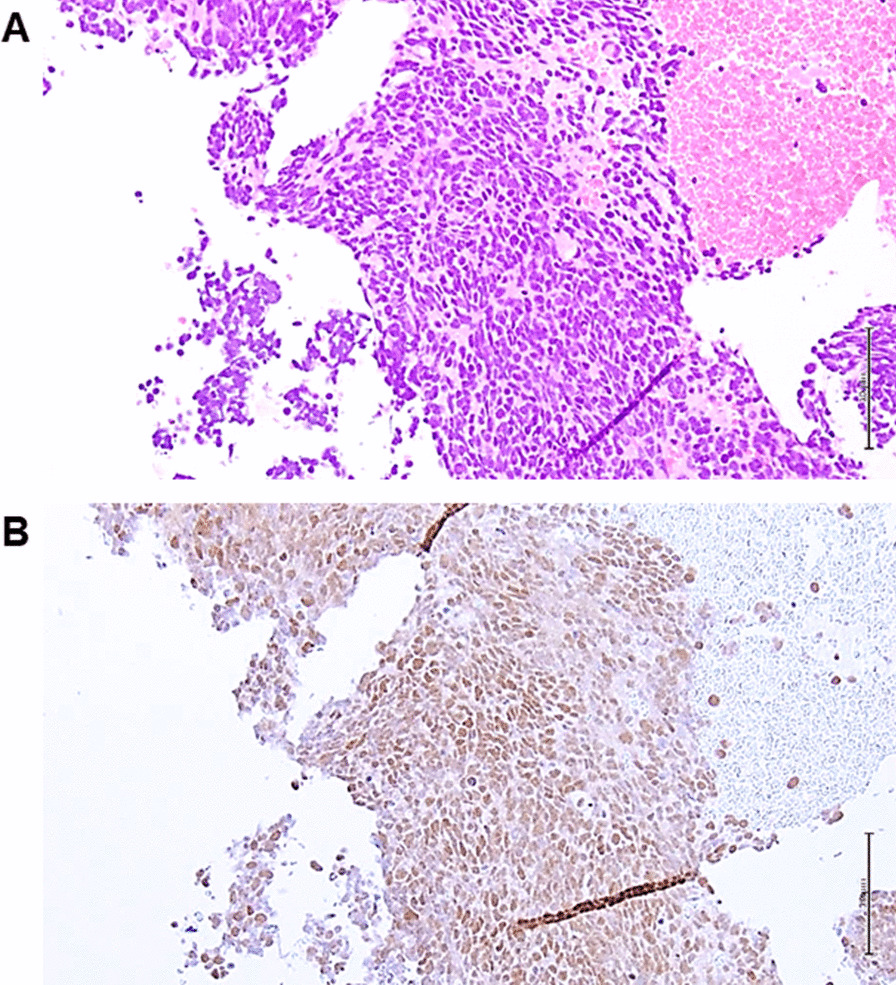
Mediastinal lymph node resected during bronchoscopy. Hematoxylin and eosin staining (**A**, ×100) and TIF1-γ immunostaining (**B**, ×100) at the time of diagnosis of lung cancer. TIF1-γ staining was strongly positive in the nucleus of tumor cells. Scale bar = 100 μm. The sections were observed with a microscope: BX53 (OLYMPUS, Tokyo, Japan), lenses: UPLNFL10×2 (OLYMPUS, Tokyo, Japan), a camera: DP22 (OLYMPUS, Tokyo Japan), and a photo system: DP2-SAL (OLYMPUS, Tokyo Japan)

A 67-year-old man with a smoking history of 55 pack-years was diagnosed with small cell lung cancer of clinical T4N3M0 stage IIIB/limited disease and received systemic chemotherapy with carboplatin and etoposide. After one course of treatment, tumor size increased, and we clinically judged the enlargement as progressive disease. Although radiation therapy was started, irradiation was terminated at 22 Gy in the middle of therapy due to a significant decrease in performance status. However, a significant decrease in tumor size was observed 4 weeks after the termination of irradiation (Fig. 1). He had no relevant medical, familial, or psycho-social history, including genetic information. However, a rash developed mainly in the precordial region and spread to the entire body (Additional file 1: Figure S1A–D) around that time. Fever, myalgia symptoms and a marked elevation in the levels of serum muscle enzymes were observed. The development of inflammatory myositis, such as polymyositis, was considered. His serum was positive for antinuclear antibodies (1:320, speckled pattern) but negative for myositis-related antibodies except TIF1-γ Ab (133 index, MESACAP Reference < 32 index). The patient was finally diagnosed as having dermatomyositis with TIF1-γ Ab. Intravenous immunoglobulin (20 g/day for 5 days) and methylprednisolone pulse therapy (1000 mg/day for 3 days) was administered, and the symptoms gradually improved with a decrease in the TIF1-γ Ab titer (111 and 84 indices at one and three months after the development of dermatomyositis, respectively). No adverse or unanticipated events occurred. Immunohistochemical analysis showed that cancer cells in the mediastinal lymph nodes that were resected during bronchoscopy at the time of diagnosis (Fig. 2A) were positive for the TIF1-γ antigen (Fig. 2B). In addition, the TIF1-γ Ab level in the serum that was collected one month before admission was within the normal limit (6 index).

## Discussion and conclusions

TIF1-γ was first reported by Venturini et al. in 1999 [4], and is one of the four TIF1 family members, which include TIF1α, TIF1β, TIF1-γ, and TIF1δ. Normal tissues, such as the skeletal muscle and skin, have been reported to express the TIF1-γ protein, which is involved in transcriptional regulation, cell proliferation, apoptosis, and carcinogenesis [1, 5]. Regarding cancers, although TIF1-γ is reported to act as both a tumor suppressor and tumor promotor depending on the cancer type [6], it is considered to be a tumor suppressor in most tumors, such as non-small cell lung cancer [7]. TIF1-γ Ab is an autoantibody that directly reacts with TIF1 and is frequently found in adult dermatomyositis patients with cancer [1, 8]. Interestingly, it has been reported that cancer in patients with TIF1-γ Ab positivity is exclusively diagnosed within 3 years before or 2.5 years following the diagnosis of myositis [9]. In addition, Aritomi et al. reported a small cell lung cancer patient with TIF1-γ Ab positivity who developed dermatomyositis after successful chemoradiotherapy [10]. Although the clinical course of Anitomi’s case is similar to that of our patient, their patient was already positive for TIF1-γ Ab. The exact mechanisms underlying the pathological condition in dermatomyositis complicated by malignant tumor have not been clarified; however, the mutated TIF1-γ protein in the tumor tissue may become the target of an autoimmune reaction that leads to the production of TIF1-γ Ab. In addition, the overexpression of self-antigens in tumor cells leads to abnormal processing and cleavage of antigenic proteins. Epitopes that are not normally exposed are induced and affect the immune system, disrupting immune tolerance and inducing TIF1-γ Ab production and autoimmunity to muscle and other tissues. It has been reported that in patients with TIF1-γ Ab positivity, TIF1-γ is highly expressed in the tumor, muscle and skin, and gene alterations such as mutation of the TIF1 gene and loss of heterozygosity are increased in the tumor [8, 11]. Moreover, TIF1-γ forms a complex with Smad 2/3, which induces Smad 2/3-dependent differentiation of Fox3-positive regulatory T cells and natural killer T cells. In the present case, the TIF1-γ protein was present in the nucleus of tumor cells in mediastinal lymph node tissue collected before the onset of dermatomyositis. Although we did not analyze whether the TIF1-γ protein was mutated, it is possible that the decrease in the tumor burden induced by treatment caused the release and exposure of a great amount of the TIF1-γ protein, failure of immune tolerance, induction of anti-TIF1-γ antibody production, and reaction of anti-TIF1-γ antibody with the TIF1-γ protein, which is expressed in the skin and muscle, resulting in the development of dermatomyositis. The finding of TIF1-γ Ab seroconversion just before the development of dermatomyositis in the present case supports our hypothesis. This case report has some limitations. First, the results in this case report are from the sole case. Second, we did not confirm an increase in the TIF1-γ protein level in the serum after the significant decrease in tumor size in the patient. Third, the presence of a mutated TIF1-γ protein in the tumor tissue was not analyzed. Finally, we did not compare the levels of the TIF1-γ protein in the serum and tumor tissues between patients with small cell carcinoma with and without dermatomyositis development. However, to our knowledge, this is the first report of TIF1-γ Ab seroconversion during chemotherapy followed by the development of dermatomyositis in a patient with lung cancer. The findings of the present case shed light on the mechanism by which anti-transcriptional intermediary factor 1-γ antibody was produced and the pathogenesis of cancer-associated myositis.

## Supplementary Information

**Additional file 1: Figure S1**. Skin findings at the onset of dermatomyositis. Erythema was observed on the face, cheeks, hair, and pinna (A). Extensive erythema, which exhibited ulceration in the central area, was found in the left precordial area (B) and on the back (C). Periungual inflammation, hemorrhagic spots, erythema and ulcers mainly on the back of the hand were observed (D).

## Data Availability

All data are contained within the manuscript.

## Abbreviations

CAM: Cancer-associated myositis
TIF1-γ: Transcriptional intermediary factor 1γ
TIF1-γ Ab: Anti-transcriptional intermediary factor 1γ antibody
