# Supplementary figures and images for: Development of dermatomyositis after anti-transcriptional intermediary factor 1-γ antibody seroconversion during treatment for small cell lung cancer
Source: BMC Pulm Med. 2022 May 12;22:191. doi: 10.1186/s12890-022-01974-4 (PMC9097056; doi:10.1186/s12890-022-01974-4)

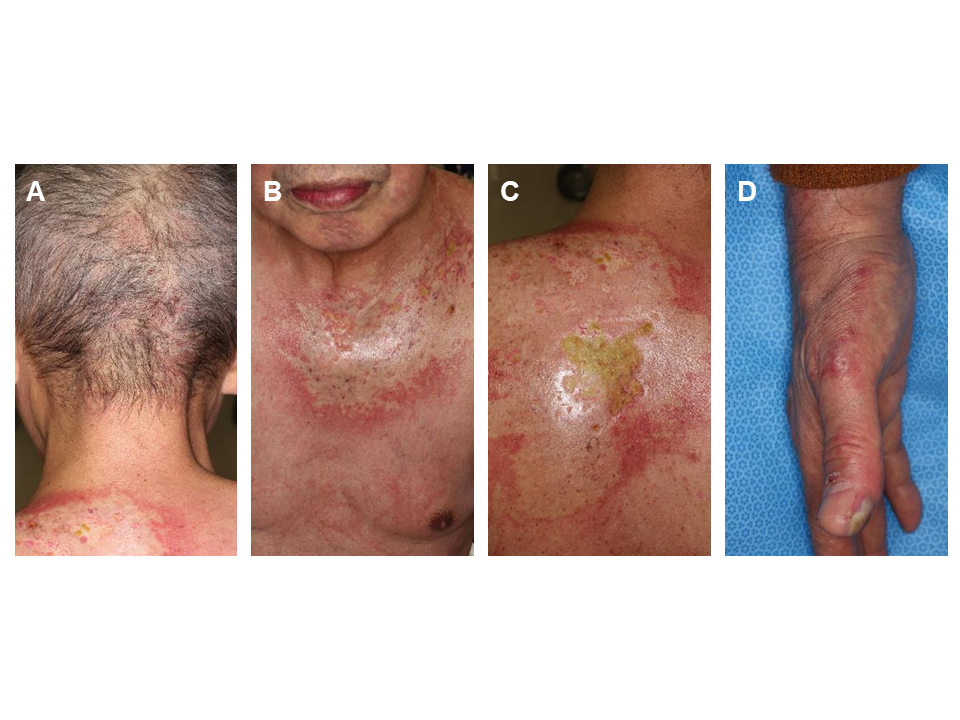

Supplement: Supplementary file 1 — Additional file 1: Figure S1. Skin findings at the onset of dermatomyositis. Erythema was observed on the face, cheeks, hair, and pinna (A). Extensive erythema, which exhibited ulceration in the central area, was found in the left precordial area (B) and on the back (C). Periungual inflammation, hemorrhagic spots, erythema and ulcers mainly on the back of the hand were observed (D). [file 12890_2022_1974_MOESM1_ESM.tif]
